# Association between dietary fiber intake and all-cause and CVD-caused mortality among heart failure survivors: a cohort study from the NHANES database

**DOI:** 10.3389/fcvm.2024.1406511

**Published:** 2025-04-10

**Authors:** Shulin Wang, Yun Ruan, Yuliang Zhang

**Affiliations:** Department of Cardiovascular Medicine, The Affiliated Qingyuan Hospital (Qingyuan People’s Hospital), Guangzhou Medical University, Qingyuan, Guangdong, China

**Keywords:** dietary fiber, heart failure, all-cause mortality, CVD-caused mortality, NHANES

## Abstract

**Aim:**

Heart failure (HF) is a severe manifestation or late stage of various heart diseases. As an anti-inflammatory nutrient, dietary fiber has been shown to be associated with the progression and prognosis of cardiovascular diseases (CVDs). However, little is known about the relationship between dietary fiber intake and mortality in HF survivors. This study evaluated the association between dietary fiber intake and all-cause and CVD-caused mortality among HF survivors.

**Methods:**

Data for the study were extracted from the National Health and Nutrition Examination Survey 1999–2018. Dietary fiber intake information was obtained by a 24-h dietary recall interview. Death outcomes were ascertained by linkage to National Death Index records through 31 December 2019. Covariates, including sociodemographic, lifestyle, disease history, and laboratory data, were extracted from the database. The weighted univariate and multivariate Cox proportional hazard models were utilized to explore the association between dietary fiber intake and mortality among HF survivors, with hazard ratios and 95% confidence intervals. Further stratified analyses were performed to explore this association based on age, gender, a history of diabetes and dyslipidemia, and duration of HF.

**Results:**

A total of 1,510 patients were included. Up to 31 December 2019, 859 deaths had occurred over a mean follow-up of 70.00 months. After multivariable adjustment, a higher dietary fiber intake was associated with a lower risk of all-cause and CVD-caused mortality in HF survivors, especially in male patients, those aged <60 years old, and those with a history of diabetes and dyslipidemia.

**Conclusion:**

Among HF survivors, higher dietary fiber intake levels may be associated with a good health outcome. More large-scale prospective cohort studies are needed to further explore this benefit relationship.

## Introduction

Globally, the prevalence of heart failure (HF) has been increasing over the past few decades. HF is a multi-faceted syndrome characterized by the inability of the heart to pump enough blood and oxygen to support the metabolic demands of other organs and affects approximately 64 million patients worldwide (1). HF is associated with increased morbidity and mortality and places a substantial burden on the healthcare system (2). The total medical costs of HF patients are expected to rise from 20.9 million dollars in 2012 to 53.1 million dollars in 2030 in the USA (3). According to a previous study, the 10-year survival of HF was only 34.9% (4). The proactive prevention of HF is of great significance to improve patient outcomes and reduce the economic burden.

Heart failure is recognized as the final stage in the progression of cardiovascular disease (5). Studies using nutritional strategies based on dietary fiber intake have been proven to be effective for the prevention and treatment of CVDs (6). Several studies reported that a higher dietary fiber intake was related to lower all-cause and CVD mortality among non-CVD populations (7–9). Thus far, evidence on the association between dietary fiber intake and mortality among the CVD population has been limited. A prospective cohort study reported that in patients who survived a myocardial infarction (MI), a greater intake of dietary fiber after an MI was inversely associated with all-cause mortality (10). Another prospective cohort study suggested that dietary fiber derived from grains may be beneficial for slowing the progression of coronary atherosclerosis in postmenopausal women (11). However, the association between dietary fiber intake and all-cause and CVD-caused mortality among patients who have survived HF remains unclear.

In this work, we explore the association between dietary fiber intake and all-cause and CVD-caused mortality among patients who survived HF, using data from the National Health and Nutrition Examination Survey (NHANES) database. This study could lead to a better understanding of the role of dietary fiber in improving the prognosis of HF patients and potentially provides reliable preventive strategies to improve the health outcomes of HF patients.

## Methods

### Study design and participants

The data for this cohort study were extracted from the NHANES 1999–2018 database. NHANES recruited a representative sample of civilian, community-dwelling members using a complex, multistage design every 2 years, and the primary objective of the study was to assess the health and nutritional status of adults and children in the USA (12). The survey was conducted by the National Center for Health Statistics (NCHS), a part of the Centers for Disease Control and Prevention (CDC). NHANES is a publicly available dataset and was approved by the NCHS Ethics Board, and all participants provided written informed consent.

In this study, data from 1,906 participants aged ≥18 years and diagnosed with HF were extracted from the NHANES 1999–2018. HF was self-reported with “Yes” for the following question about HF in the questionnaire at a Mobile Examination Center (MEC): “Has a doctor ever told you that you had HF?” (13). Of the participants that were initially extracted, 308 were missing dietary fiber intake information, 87 had an extreme energy intake (male: <800 or >6,000 kcal/day; female: <600 or >4,000 kcal/day) (14), and 1 participant was missing survival data. These participants were excluded. Thus, finally, 1,510 eligible HF patients were included for further analyses.

### Dietary fiber intake assessment

Total dietary fiber intake was obtained through a 24-h dietary recall interview by a trained interviewer at an NHANES MEC. All the participants were asked to recall the type and amount of food and beverages they consumed in the past 24 h. Dietary fiber intake was calculated according to the US Department of Agriculture (USDA) Food and Nutrient Database for Dietary Studies (15). In this study, total dietary fiber intake was divided into three categories: Q1 (8.8 g), Q2 (13.3 g), and Q3 (19.1 g).

### Outcomes and follow-up

The outcomes of this study were all-cause mortality and CVD-caused mortality among HF patients. Mortality data were confirmed through the National Death Index, contact with next of kin, or via the postal system (16, 17). Information regarding the cause of death was collected by a review of medical records by an experienced physician. All-cause mortality among HF patients included mortalities caused by a malignant tumor, CVDs, respiratory disease, Alzheimer's disease, diabetes, nephropathy-related diseases, accidental death, and other causes (18). CVD was defined as the composite of incident non-fatal myocardial infarction, fatal coronary heart disease (CHD), and fatal and non-fatal strokes. A non-fatal myocardial infarction and stroke were confirmed using World Health Organization criteria (19) and the National Survey of Stroke criteria (20), respectively.

### Potential covariates

The demographic, lifestyle, disease history, and laboratory data of the participants were extracted from the NHANES database. Smoking status was assessed by the question “Have you smoked at least 100 cigarettes in your life” (answered “yes” or “no”) (21). Participants who answered negatively to the question “Have you had at least 12 alcoholic drinks in 1 year?” were defined as never drinkers. Participants who answered the question “On average, how many times per week did you drink alcohol in the past 12 months?” with “less than once per week” were defined as occasional drinkers and “more than once per week” were defined as regular drinkers (22). Physical activity was expressed as the metabolic equivalent of task (MET) and calculated as follows: physical activity (MET min/week) = recommended MET × exercise time for corresponding activities (min/day) × the number of exercise days per week (days) (23). The history of angina and stroke was adapted from the health questionnaire. Hypertension was defined as systolic blood pressure (SBP) ≥130 mmHg, diastolic blood pressure (DBP) ≥80 mmHg, self-reported, or taking hypotensive drugs (24). Dyslipidemia was defined as total cholesterol (TC) ≥200 mg/dl, triglyceride (TG) ≥150 mg/dl, low-density lipoprotein cholesterol (LDL-C) ≥130 mg/dl, high-density lipoprotein cholesterol (HDL-C) ≤40 mg/dl, or self-reported (25). Diabetes was defined according to the following criteria: self-report of a diabetes diagnosis by a clinician or health professional, glycated hemoglobin (HbA1c) ≥6.5%, fasting glucose ≥126 mg/dl, or 2 h oral glucose tolerance test (OGTT) ≥200 mmol/L (26). Chronic kidney disease (CKD) was defined as estimated glomerular filtration rate <60 ml/min/1.73 m^2^ and eGFR was determined using the Chronic Kidney Disease-Epidemiology Collaboration (CKD-EPI) equation (27). Patients with a urine albumin-to-creatinine ratio (UACR) ≥30 mg/g were also defined as having CKD (28). Depression was evaluated using the patient health questionnaire (PHQ-9) and a PHQ-9 score ≥10 was defined as being depressed (29). Body mass index (BMI) was classified into four levels: underweight (<18.5 kg/m^2^), normal (18.5–24.9 kg/m^2^), overweight (25–19.9 kg/m^2^), and obese (≥30 kg/m^2^) (30).

### Statistical analysis

All statistical analyses were conducted by SDMVPSU, SDMVSTRA, and WTDRD1. SDMVSTRA was the confidence interval (CI) that was applied to assess the reliability of an estimate. WTDRD1 refers to the weight for a dietary day of one 2-year sample. SDMVPSU means that the masked variance unit pseudo-substrate is sdmvstra, and the masked variance unit pseudo-primary unit is sdmvpsu.

Continuous data were expressed as mean and standard error (SE) and the weighted *t*-test was used for comparisons between two groups. Qualitative data were expressed as the number and proportion [*n* (%)], and *χ*^2^ was used for comparison between the two groups. Sensitivity analyses were performed to ascertain whether the results were different before and after imputation (Supplementary Table S1). The weighted Cox proportional-hazards regression models were used to explore the association between dietary fiber intake and all-cause mortality and CVD-caused mortality among HF patients, with hazard ratios (HRs) and 95% CIs. Model 1 was a crude model without adjusting for covariates. Model 2 was adjusted for age, race, education level, marital status, physical activity, stroke, hypertension, diabetes, CKD, energy, hemoglobin, uric acid, WBC, albumin, and drugs for CVDs. Subgroup analyses were further conducted to explore the association based on age, gender, and history of diabetes and dyslipidemia. Two-sided *P* < 0.05 was considered as statistically significant.

## Results

### Baseline characteristics

**Figure 1:**
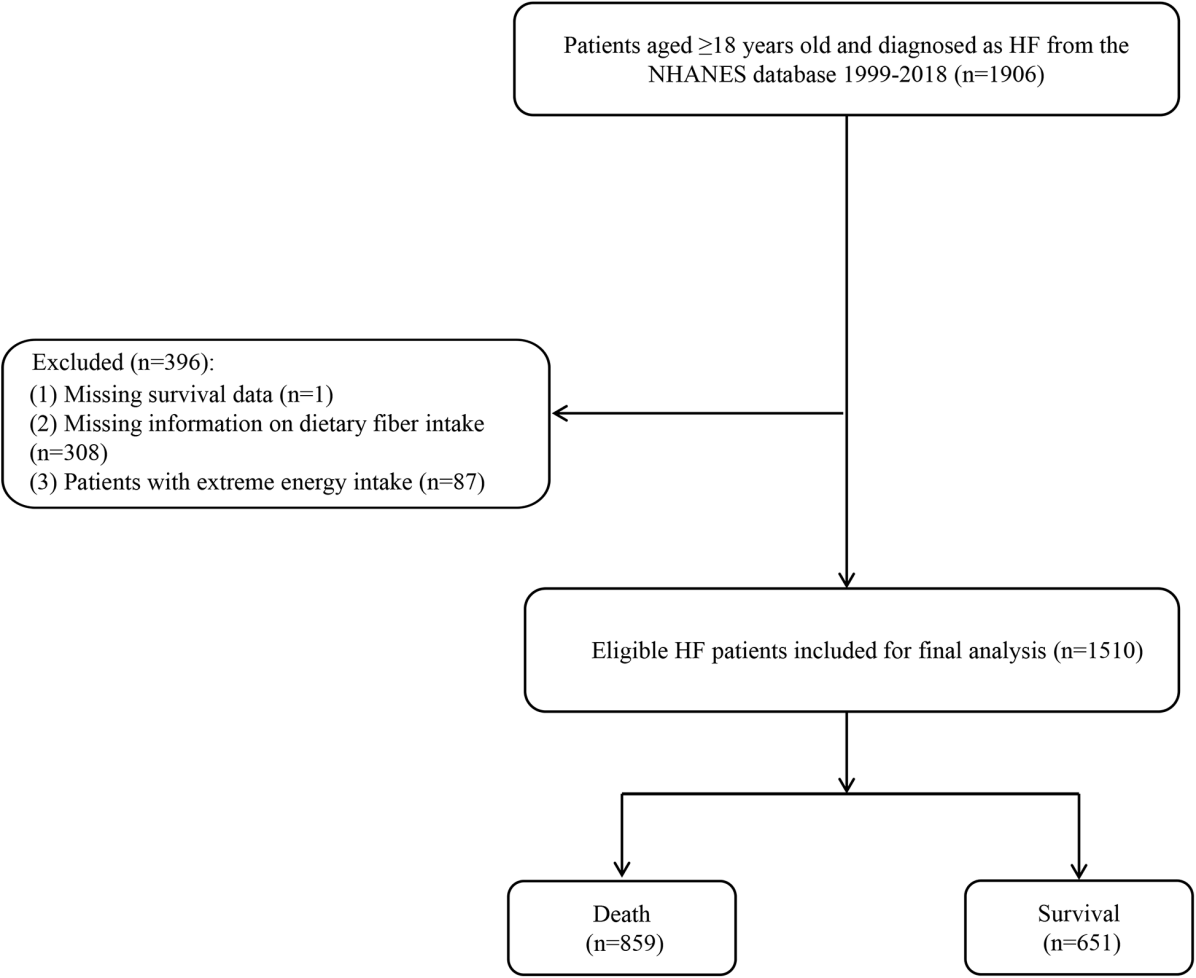
Flow chart of the screening process for the selection of participants in NHANES 1999–2018.

A flow chart of the population screening process is shown in Figure 1. In total, 1,510 HF patients were included with a mean weighted age of 66.10 (0.49) years old and 53.79% were men. The characteristics of the included participants are shown in Tables 1, 2. Notably, the proportion of those who had a high dietary fiber intake in the survival group was significantly higher than in the all-cause and CVD-caused mortality group. Differences were found in age, race, BMI, education, physical activity, energy, hemoglobin, uric acid, albumin, marital status, drinking, history of hypertension, CKD, depression, family heart attack, and CVD drug use between the survival and all-cause mortality groups. For the CVD-caused mortality and survival groups, differences were found in age, BMI, energy, hemoglobin, physical activity, marital status, history of CKD, depression, family heart attack, and CVD drug use (all *P* < 0.05).

**Table 1:** Characteristics of HF survivors with all-cause mortality.

Variable	Total (*n* = 1,510)	Survival (*n* = 651)	All-cause mortality (*n* = 859)	Statistics	*P*
Age, years, mean (SE)	66.10 (0.49)	61.26 (0.77)	70.24 (0.54)	*t* = −9.59	<0.001
Gender, *n* (%)				*χ*^2^ = 0.447	0.504
Men	850 (53.79)	356 (55.13)	494 (52.64)		
Women	660 (46.21)	295 (44.87)	365 (47.36)		
BMI, kg/m^2^, *n* (%)				χ^2^ = 18.366	<0.001
<18.5	16 (0.89)	7 (0.87)	9 (0.92)		
18.5–25	268 (16.95)	84 (12.72)	184 (20.56)		
25–30	461 (28.98)	188 (26.40)	273 (31.17)		
≥30	765 (53.17)	372 (60.02)	393 (47.34)		
Race, *n* (%)				χ^2^ = 14.150	<0.001
White	827 (73.19)	298 (67.65)	529 (77.91)		
Black	355 (13.80)	180 (16.16)	175 (11.79)		
Unknown	328 (13.01)	173 (16.19)	155 (10.30)		
Education level, *n* (%)				χ^2^ = 12.742	<0.001
High school and below	967 (59.01)	379 (51.95)	588 (65.02)		
Above high school	543 (40.99)	272 (48.05)	271 (34.98)		
PIR, *n* (%)				χ^2^ = 0.081	0.960
<1.3	530 (28.98)	238 (28.66)	292 (29.25)		
≥1.3	850 (63.35)	354 (63.46)	496 (63.25)		
Unknown	130 (7.67)	59 (7.88)	71 (7.50)		
Marital status, *n* (%)				χ^2^ = 31.019	<0.001
Married	708 (49.81)	310 (52.88)	398 (47.18)		
Widowed	389 (23.13)	120 (15.12)	269 (29.95)		
Divorced	200 (13.06)	98 (13.42)	102 (12.74)		
Separated	56 (3.14)	32 (3.48)	24 (2.85)		
Never married	109 (7.91)	62 (11.22)	47 (5.09)		
Living with partner	48 (2.96)	29 (3.88)	19 (2.18)		
Smoking, *n* (%)				χ^2^ = 0.117	0.732
No	586 (36.61)	263 (37.18)	323 (36.12)		
Yes	924 (63.39)	388 (62.82)	536 (63.88)		
Drinking, *n* (%)				χ^2^ = 10.608	0.005
Never drinker	595 (37.78)	262 (36.06)	333 (39.24)		
Regular drinker	259 (18.98)	129 (23.79)	130 (14.87)		
Occasional drinker	656 (43.24)	260 (40.15)	396 (45.88)		
Physical activity, MET min/week, mean (SE)				χ^2^ = 88.356	<0.001
<450	744 (46.20)	353 (49.07)	391 (43.75)		
≥450	387 (27.93)	244 (41.00)	143 (16.79)		
Unknown	379 (25.87)	54 (9.93)	325 (39.46)		
Angina, *n* (%)				χ^2^ = 0.766	0.381
No	1,119 (72.23)	499 (73.90)	620 (70.80)		
Yes	391 (27.77)	152 (26.10)	239 (29.20)		
Heart attack, *n* (%)				χ^2^ = 0.888	0.346
No	832 (55.14)	382 (57.26)	450 (53.33)		
Yes	678 (44.86)	269 (42.74)	409 (46.67)		
Stroke, *n* (%)				χ^2^ = 1.782	0.182
No	1,202 (80.10)	542 (82.06)	660 (78.42)		
Yes	308 (19.90)	109 (17.94)	199 (21.58)		
Hypertension, *n* (%)				χ^2^ = 6.114	0.013
No	62 (5.54)	37 (7.90)	25 (3.52)		
Yes	1,448 (94.46)	614 (92.10)	834 (96.48)		
Dyslipidemia, *n* (%)				χ^2^ = 0.809	0.369
No	269 (15.82)	101 (14.53)	168 (16.93)		
Yes	1,241 (84.18)	550 (85.47)	691 (83.07)		
Diabetes, *n* (%)				χ^2^ = 1.879	0.170
No	794 (56.14)	356 (58.86)	438 (53.82)		
Yes	716 (43.86)	295 (41.14)	421 (46.18)		
CKD, *n* (%)				χ^2^ = 60.689	<0.001
No	906 (64.76)	480 (78.38)	426 (53.16)		
Yes	485 (29.14)	135 (17.24)	350 (39.27)		
Unknown	119 (6.10)	36 (4.38)	83 (7.57)		
Depression, *n* (%)				χ^2^ = 15.156	<0.001
No	1,334 (87.46)	546 (83.12)	788 (91.16)		
Yes	176 (12.54)	105 (16.88)	71 (8.84)		
Family history of heart attack, *n* (%)				χ^2^ = 5.173	0.023
No	1,234 (80.37)	503 (76.65)	731 (83.54)		
Yes	276 (19.63)	148 (23.35)	128 (16.46)		
Duration of HF, years				χ^2^ = 1.958	0.376
<10	929 (62.04)	405 (61.10)	524 (62.83)		
≥10	555 (36.33)	239 (37.77)	316 (35.10)		
Unknown	26 (1.64)	7 (1.13)	19 (2.07)		
Energy, kcal, mean (SE)	1,849.02 (30.86)	1,974.77 (55.06)	1,741.87 (33.72)	*t* = 3.51	<0.001
Hemoglobin, g/dl, mean (SE)	13.80 (0.07)	14.02 (0.10)	13.61 (0.09)	*t* = 3.23	0.002
ALT, U/L, mean (SE)	26.01 (1.85)	26.84 (1.88)	25.31 (3.03)	*t* = 0.43	0.666
AST, U/L, mean (SE)	27.06 (1.00)	26.75 (1.40)	27.32 (1.41)	*t* = −0.29	0.774
Uric acid, mg/dl, mean (SE)	6.45 (0.07)	6.21 (0.10)	6.65 (0.10)	*t* = −3.19	0.002
WBC, SI, mean (SE)	7.75 (0.08)	7.59 (0.11)	7.88 (0.13)	*t* = −1.69	0.094
Albumin, g/L, mean (SE)	40.91 (0.13)	41.33 (0.23)	40.55 (0.15)	*t* = 2.76	0.006
Drug for CVDs, *n* (%)				χ^2^ = 16.090	<0.001
No	666 (45.02)	333 (52.63)	333 (38.54)		
Yes	844 (54.98)	318 (47.37)	526 (61.46)		
Dietary fiber, g, mean (SE)	14.74 (0.28)	15.82 (0.45)	13.82 (0.33)	*t* = 3.62	<0.001
Dietary fiber, g, *n* (%)				χ^2^ = 16.712	<0.001
1: <8.80	408 (25.00)	170 (22.73)	238 (26.93)		
2: 8.80–13.30	384 (24.97)	152 (22.03)	232 (27.48)		
3: 13.30–19.10	359 (25.00)	152 (23.90)	207 (25.94)		
4: ≥19.10	359 (25.03)	177 (31.34)	182 (19.65)		

*t*, *t*-test; *χ*^2^, chi-square; SE, standard error; HF, heart failure; BMI, body mass index; PIR, poverty-to-income ratio; MET, metabolic equivalent of task; CKD, chronic kidney disease; ALT, alanine aminotransferase; AST, aspartate transaminase; WBC, white blood cell; CVDs, cardiovascular diseases.

**Table 2:** Characteristics of HF survivors with CVD-caused mortality.

Variable	Total (*n* = 1,510)	Survival (*n* = 1,143)	CVD-caused mortality (*n* = 367)	Statistics	*P*
Age, years, mean (SE)	66.10 (0.49)	64.80 (0.58)	70.59 (0.72)	t = −6.55	<0.001
Gender, *n* (%)				χ^2^ = 0.542	0.462
Men	850 (53.79)	640 (54.36)	210 (51.82)		
Women	660 (46.21)	503 (45.64)	157 (48.18)		
BMI, kg/m^2^, *n* (%)				χ^2^ = 8.071	0.045
<18.5	16 (0.89)	12 (0.87)	4 (0.96)		
18.5–25	268 (16.95)	194 (15.95)	74 (20.41)		
25–30	461 (28.98)	344 (27.94)	117 (32.54)		
≥30	765 (53.17)	593 (55.23)	172 (46.09)		
Race, *n* (%)				χ^2^ = 2.152	0.341
White	827 (73.19)	605 (72.14)	222 (76.81)		
Black	355 (13.80)	280 (14.22)	75 (12.37)		
Unknown	328 (13.01)	258 (13.64)	70 (10.82)		
Education level, *n* (%)				χ^2^ = 1.679	0.195
High school and below	967 (59.01)	720 (57.78)	247 (63.26)		
Above high school	543 (40.99)	423 (42.22)	120 (36.74)		
PIR, *n* (%)				χ^2^ = 2.586	0.274
<1.3	530 (28.98)	409 (29.94)	121 (25.67)		
≥1.3	850 (63.35)	635 (62.19)	215 (67.33)		
Unknown	130 (7.67)	99 (7.87)	31 (7.00)		
Marital status, *n* (%)				χ^2^ = 11.176	0.048
Married	708 (49.81)	531 (49.54)	177 (50.72)		
Widowed	389 (23.13)	276 (21.27)	113 (29.52)		
Divorced	200 (13.06)	165 (14.23)	35 (9.01)		
Separated	56 (3.14)	47 (3.52)	9 (1.83)		
Never married	109 (7.91)	85 (8.45)	24 (6.04)		
Living with partner	48 (2.96)	39 (2.99)	9 (2.88)		
Smoking, *n* (%)				χ^2^ = 2.572	0.109
No	586 (36.61)	429 (35.31)	157 (41.08)		
Yes	924 (63.39)	714 (64.69)	210 (58.92)		
Drinking, *n* (%)				χ^2^ = 1.689	0.430
Never drinker	595 (37.78)	454 (37.60)	141 (38.41)		
Regular drinker	259 (18.98)	200 (19.86)	59 (15.92)		
Occasional drinker	656 (43.24)	489 (42.54)	167 (45.67)		
Physical activity, MET min/week, mean (SE)				χ^2^ = 30.788	<.001
<450	744 (46.20)	588 (47.41)	156 (42.04)		
≥450	387 (27.93)	326 (31.06)	61 (17.17)		
Unknown	379 (25.87)	229 (21.54)	150 (40.79)		
Angina, *n* (%)				χ^2^ = 0.342	0.558
No	1,119 (72.23)	854 (72.74)	265 (70.45)		
Yes	391 (27.77)	289 (27.26)	102 (29.55)		
Heart attack, *n* (%)				χ^2^ = 1.822	0.177
No	832 (55.14)	647 (56.43)	185 (50.68)		
Yes	678 (44.86)	496 (43.57)	182 (49.32)		
Stroke, *n* (%)				χ^2^ = 0.749	0.387
No	1,202 (80.10)	920 (80.68)	282 (78.09)		
Yes	308 (19.90)	223 (19.32)	85 (21.91)		
Hypertension, *n* (%)				χ^2^ = 1.879	0.170
No	62 (5.54)	53 (6.15)	9 (3.45)		
Yes	1,448 (94.46)	1,090 (93.85)	358 (96.55)		
Dyslipidemia, *n* (%)				χ^2^ = 0.052	0.820
No	269 (15.82)	205 (15.95)	64 (15.38)		
Yes	1,241 (84.18)	938 (84.05)	303 (84.62)		
Diabetes, *n* (%)				χ^2^ = 3.564	0.059
No	794 (56.14)	610 (57.90)	184 (50.08)		
Yes	716 (43.86)	533 (42.10)	183 (49.92)		
CKD, *n* (%)				*χ*^2^ = 31.840	<0.001
No	906 (64.76)	727 (68.93)	179 (50.41)		
Yes	485 (29.14)	334 (25.31)	151 (42.32)		
Unknown	119 (6.10)	82 (5.76)	37 (7.26)		
Depression, *n* (%)				*χ*^2^ = 4.505	0.034
No	1,334 (87.46)	997 (86.26)	337 (91.60)		
Yes	176 (12.54)	146 (13.74)	30 (8.40)		
Family history of heart attack, *n* (%)				*χ*^2^ = 12.938	<0.001
No	1,234 (80.37)	911 (77.96)	323 (88.68)		
Yes	276 (19.63)	232 (22.04)	44 (11.32)		
Duration of HF, years				*χ*^2^ = 1.783	0.410
<10	929 (62.04)	709 (61.95)	220 (62.35)		
≥10	555 (36.33)	417 (36.66)	138 (35.17)		
Unknown	26 (1.64)	17 (1.39)	9 (2.48)		
Energy, kcal, mean (SE)	1,849.02 (30.86)	1,894.15 (36.38)	1,693.57 (47.45)	*t* = 3.41	<0.001
Hemoglobin, g/dl, mean (SE)	13.80 (0.07)	13.88 (0.07)	13.51 (0.13)	*t* = 2.59	0.011
ALT, U/L, mean (SE)	26.01 (1.85)	27.13 (2.38)	22.17 (0.77)	*t* = 1.97	0.051
AST, U/L, mean (SE)	27.06 (1.00)	27.43 (1.25)	25.80 (0.67)	*t* = 1.21	0.230
Uric acid, mg/dl, mean (SE)	6.45 (0.07)	6.40 (0.08)	6.61 (0.15)	*t* = −1.30	0.194
WBC, SI, mean (SE)	7.75 (0.08)	7.79 (0.11)	7.60 (0.18)	*t* = 0.87	0.383
Albumin, g/L, mean (SE)	40.91 (0.13)	40.94 (0.17)	40.77 (0.24)	*t* = 0.56	0.574
Drug for CVDs, *n* (%)				*χ*^2^ = 14.748	<0.001
No	666 (45.02)	543 (48.24)	123 (33.93)		
Yes	844 (54.98)	600 (51.76)	244 (66.07)		
Dietary fiber, g, mean (SE)	14.74 (0.28)	15.01 (0.32)	13.83 (0.51)	*t* = 1.98	0.049
Dietary fiber, g, *n* (%)				χ^2^ = 6.545	0.088
1: <8.80	408 (25.00)	305 (23.58)	103 (29.89)		
2: 8.80–13.30	384 (24.97)	283 (24.61)	101 (26.21)		
3: 13.30–19.10	359 (25.00)	276 (25.18)	83 (24.38)		
4: ≥19.10	359 (25.03)	279 (26.63)	80 (19.52)		

*T*, *t*-test; *χ*^2^, chi-square; SE, standard error; HF, heart failure; CVD, cardiovascular disease; BMI, body mass index; PIR, poverty-to-income ratio; MET, metabolic equivalent of task; CKD, chronic kidney disease; ALT, alanine aminotransferase; AST, aspartate transaminase; WBC, white blood cell.

### Relationship between dietary fiber intake and mortality among HF patients

We employed two weighted logistic regression models to investigate the association between dietary fiber intake and all-cause and CVD-caused mortality, as presented in Tables 3, 4. After adjusting for all variables, compared to those in the lowest quartile of dietary fiber intake (<8.80 g), all-cause mortality among those in the highest quartile of dietary fiber intake (≥19.10 g) was reduced by 32% (95% CI: 0.51–0.90); CVD-caused mortality among those in the highest quartile of dietary fiber intake (≥19.10 gm) was reduced by 40% (95% CI: 0.40–0.88) (all *P* < 0.05).

**Table 3:** Association between dietary fiber intake and all-cause mortality among HF survivors.

Variables	Model 1		Model 2	
	HR (95% CI)	*P*	HR (95% CI)	*P*
Dietary fiber, g				
<8.80	Ref		Ref	
8.80–13.30	1.17 (0.94–1.46)	0.159	0.89 (0.69–1.16)	0.380
13.30–19.10	1.01 (0.76–1.34)	0.962	0.93 (0.70–1.23)	0.609
≥19.10	0.76 (0.59–0.97)	0.029	0.68 (0.51–0.90)	0.007

Ref: reference; HR: hazard ratio; CI: confidence interval; HF: heart failure.

Model 1: crude model. Model 2: adjusted for age, race, education level, marital status, physical activity, stroke, hypertension, diabetes, CKD, energy, hemoglobin, uric acid, WBC, albumin, and drugs for CVDs.

**Table 4:** Association between dietary fiber and CVD-caused mortality among HF survivors.

Variable	Model 1		Model 2	
	HR (95% CI)	*P*	HR (95% CI)	*P*
Dietary fiber, g				
<8.80	Ref		Ref	
8.80–13.30	0.99 (0.69–1.42)	0.953	0.75 (0.51–1.12)	0.163
13.30–19.10	0.85 (0.57–1.25)	0.400	0.76 (0.50–1.16)	0.204
≥19.10	0.67 (0.47–0.96)	0.028	0.60 (0.40–0.88)	0.010

Ref, reference; HR, hazard ratio; CI, confidence interval; CVDs, cardiovascular diseases; HF, heart failure.

Model 1: crude model. Model 2: adjusted for age, marital status, physical activity, diabetes, CKD, energy, hemoglobin, ALT, uric acid, albumin, and drugs for CVDs.

### Subgroup analyses

As depicted in Tables 5, 6, further stratified analyses were conducted to explore the association between dietary fiber intake and all-cause and CVD-caused mortality. Among HF patients who were men, aged <60 years old, and had a duration of HF ≥10 years, the relationship between the highest quartile of dietary fiber intake (≥19.10 g) and a lower risk of all-cause mortality and CVD-caused mortality was more pronounced. Among HF patients with diabetes, all-cause mortality and CVD-caused mortality among those in the highest quartile of dietary fiber intake (≥19.10 g) were reduced by 41% and 46%, respectively.

**Table 5:** Association between dietary fiber intake and all-cause mortality among HF survivors based on age, gender, and a history of diabetes and dyslipidemia.

Variable	Model 2							
	Age <60 years		Age ≥60 years		Men		Women	
	HR (95% CI)	*P*	HR (95% CI)	*P*	HR (95% CI)	*P*	HR (95% CI)	*P*
Dietary fiber, g								
<8.80	Ref		Ref		Ref		Ref	
8.80–13.30	0.69 (0.32–1.48)	0.330	1.06 (0.78–1.45)	0.688	0.71 (0.51–0.99)	0.046	1.11 (0.80–1.54)	0.540
13.30–19.10	1.01 (0.55–1.82)	0.999	1.04 (0.75–1.44)	0.831	0.74 (0.53–1.05)	0.093	1.10 (0.68–1.78)	0.704
≥19.10	0.32 (0.19–0.54)	<.001	0.89 (0.64–1.25)	0.495	0.52 (0.38–0.70)	<.001	0.82 (0.54–1.25)	0.358
Variable	Non-diabetes		Diabetes		Non-dyslipidemia		Dyslipidemia	
	HR (95% CI)	*P*	HR (95% CI)	*P*	HR (95% CI)	*P*	HR (95% CI)	*P*
Dietary fiber, g								
<8.80	Ref		Ref		Ref		Ref	
8.80–13.30	0.92 (0.66–1.30)	0.649	0.77 (0.53–1.11)	0.157	0.83 (0.53–1.30)	0.408	0.90 (0.66–1.24)	0.523
13.30–19.10	1.16 (0.78–1.71)	0.461	0.66 (0.46–0.94)	0.023	1.02 (0.62–1.69)	0.934	0.89 (0.65–1.21)	0.457
≥19.10	0.67 (0.45–0.98)	0.039	0.59 (0.40–0.87)	0.007	0.60 (0.34–1.04)	0.069	0.70 (0.52–0.94)	0.019
Variable	Duration of HF <10 years				Duration of HF ≥10 years			
	HR (95% CI)		*P*		HR (95% CI)		*P*	
Dietary fiber, g								
<8.80	Ref				Ref			
8.80–13.30	0.77 (0.54–1.09)		0.133		0.84 (0.58–1.22)		0.368	
13.30–19.10	0.83 (0.59–1.16)		0.273		0.91 (0.57–1.46)		0.705	
≥19.10	0.83 (0.59–1.16)		0.273		0.55 (0.35–0.87)		0.101	

Ref, reference; HR, hazard ratio; CI, confidence interval; HF, heart failure.

In the age subgroup: adjusted for race, education level, marital status, physical activity, stroke, hypertension, diabetes, CKD, energy, hemoglobin, uric acid, WBC, albumin, and drugs for CVDs. In the gender subgroup: adjusted for age, race, education level, marital status, physical activity, stroke, hypertension, diabetes, CKD, energy, hemoglobin, uric acid, WBC, albumin, and drugs for CVDs. In the diabetes group: adjusted for age, race, education level, marital status, physical activity, stroke, hypertension, CKD, energy, hemoglobin, uric acid, WBC, albumin, and drugs for CVDs. In the dyslipidemia subgroup: adjusted for age, race, education level, marital status, physical activity, stroke, hypertension, diabetes, CKD, energy, hemoglobin, uric acid, WBC, albumin, and drugs for CVDs. In the duration of HF group: adjusted for age, race, education level, marital status, physical activity, stroke, hypertension, diabetes, CKD, energy, hemoglobin, uric acid, WBC, albumin, and drugs for CVDs.

**Table 6:** Association between dietary fiber and CVD-caused mortality among HF survivors based on age, gender, and a history of diabetes and dyslipidemia.

Variable	Model 2							
	Age <60 years		Age ≥60 years		Men		Women	
	HR (95% CI)	*P*	HR (95% CI)	*P*	HR (95% CI)	*P*	HR (95% CI)	*P*
Dietary fiber, g								
<8.80	Ref		Ref		Ref		Ref	
8.80–13.30	0.85 (0.51–1.43)	0.540	0.91 (0.56–1.48)	0.709	0.71 (0.43–1.17)	0.177	0.93 (0.53–1.62)	0.802
13.30–19.10	0.48 (0.22–1.05)	0.064	1.03 (0.62–1.70)	0.916	0.56 (0.32–0.98)	0.041	1.21 (0.66–2.20)	0.539
≥19.10	0.31 (0.16–0.60)	<.001	0.81 (0.50–1.31)	0.393	0.51 (0.31–0.86)	0.012	0.63 (0.34–1.17)	0.142
Variables	Non-diabetes		Diabetes		Non-dyslipidemia		Dyslipidemia	
	HR (95% CI)	*P*	HR (95% CI)	*P*	HR (95% CI)	*P*	HR (95% CI)	*P*
Dietary fiber, g								
<8.80	Ref		Ref		Ref		Ref	
8.80–13.30	0.79 (0.45–1.36)	0.383	0.73 (0.45–1.20)	0.211	0.45 (0.24–0.86)	0.016	0.83 (0.53–1.31)	0.426
13.30–19.10	1.03 (0.52–2.04)	0.921	0.56 (0.34–0.94)	0.030	0.66 (0.28–1.55)	0.331	0.75 (0.47–1.20)	0.232
≥19.10	0.54 (0.29–1.01)	0.052	0.54 (0.29–0.99)	0.049	0.33 (0.14–0.76)	0.011	0.64 (0.41–0.98)	0.041
Variables	Duration of HF <10 years				Duration of HF ≥10 years			
	HR (95% CI)		*P*		HR (95% CI)		*P*	
Dietary fiber, gm								
<8.80		Ref				Ref		
8.80–13.30		0.76 (0.45–1.30)		0.313		0.63 (0.38–1.02)		0.061
13.30–19.10		0.94 (0.55–1.61)		0.831		0.36 (0.16–0.79)		0.011
≥19.10		0.80 (0.50–1.28)		0.344		0.29 (0.16–0.52)		<0.001

Ref, reference; HR, hazard ratio; CI, confidence interval; HF, heart failure.

In the age subgroup: adjusted for marital status, physical activity, diabetes, CKD, energy, hemoglobin, ALT, uric acid, albumin, and drugs for CVDs. In the gender group: adjusted for age, marital status, physical activity, diabetes, CKD, energy, hemoglobin, ALT, uric acid, albumin, and drugs for CVDs. In the diabetes group: adjusted for age, marital status, physical activity, CKD, energy, hemoglobin, ALT, uric acid, albumin, and drugs for CVDs. In the dyslipidemia subgroup: adjusted for age, marital status, physical activity, diabetes, CKD, energy, hemoglobin, ALT, uric acid, albumin, and drugs for CVDs. In the duration of HF group: adjusted for age, race, education level, marital status, physical activity, stroke, hypertension, diabetes, CKD, energy, hemoglobin, uric acid, WBC, albumin and, drugs for CVDs.

## Discussion

Among HF survivors in this retrospective cohort from the USA, we found that a high dietary fiber intake level was associated with a lower risk of all-cause and CVD-caused mortality. The stratified analyses based on age, gender, a history of diabetes and dyslipidemia, and duration of HF suggested the findings were robust. For the prognostic management of HF survivors, a high dietary fiber intake in their daily diet may yield greater benefits.

Currently, a small number of studies have focused on dietary intervention for the risk of death in patients with HF, but no consistent conclusions have been reached. Chang et al. (31) explored the association between adherence to the Mediterranean diet (aMED) with all-cause mortality in patients with HF and observed that a Mediterranean diet was not associated with mortality; however, among the aMED components, a lower red/processed meat intake was associated with higher mortality risk. Sun and Du (32) assessed the association of dietary magnesium intake and vitamin D levels with the risk of mortality in HF patients and suggested that vitamin D and magnesium levels may have a joint effect on improving the prognosis of HF patients. Dietary fiber contains a unique blend of bioactive components including resistant starches, vitamins, minerals, phytochemicals, and antioxidants (33). As a result, studies regarding their potential health benefits have received considerable attention in the last several decades. Epidemiological and clinical studies reported that the consumption of dietary fiber was inversely related to CVDs. The Zutphen study conducted by Streppel et al. suggested that a higher dietary fiber intake was associated with a lower risk of both CHD and all-cause mortality (34). Every additional 10 g of dietary fiber intake per day reduced coronary heart disease mortality by 7% and all-cause mortality by 9%. Another study from the Nurses’ Health Study documented 761 CHD cases, and, after adjusting for age and smoking, increased whole-grain intake was associated with a decreased risk of CHD (35). The consumption of whole-grain products is often associated with beneficial effects for consumer health. Dietary fiber is a vital component present in whole grains and is believed to be responsible for these health benefits (36). Two previous prospective cohort studies also examined whether the intake of whole-grain foods was related to CVD-caused morbidity and mortality (37, 38). Both studies reported consistent results that high levels of dietary fiber-rich food intake were inversely associated with cardiovascular-related morbidity and mortality.

Although the mechanism of the progression of HF has not been fully elucidated, disturbances in the metabolic and inflammatory pathways seem to play a vital role (39). The gut microbiota, including the bacteria that reside in the gastrointestinal tract, may influence these pathways (40). The interaction between the gut microbiome and the host determines key physiological processes in human metabolism, including inflammatory response, metabolic function, and disease susceptibility (41). The maintenance and improvement of the balance of intestinal flora has a positive regulatory effect on physiological processes in the body. The intestinal microbiome is largely regulated by diet, and a dietary fiber intervention can change the composition and abundance of intestinal microorganisms in the human body to a certain extent (42). Heart and intestinal failure are closely related. The current leaky gut hypothesis for HF holds that intestinal edema and barrier dysfunction caused by HF cause the translocation of intestinal flora components into the host’s circulatory system, resulting in endotoxemia and ultimately aggravating systemic inflammation (43).

To further evaluate the robustness of the results, we examined the association between dietary fiber and all-cause and CVD-caused mortality in a specific population of HF patients. After adjusting for all the covariates, we also found an inverse association between dietary fiber and all-cause and CVD-caused mortality among HF patients, especially among men and those younger than 60 years old. Among the HF survivors with a history of diabetes and dyslipidemia, a high dietary fiber intake was associated with a lower risk for all-cause and CVD-caused mortality. A previous study reported that there was a gender difference in HF (44). Except for those aged 80 years and older, the incidence of HF was lower in women than in men in all age groups. One possible reason for the higher incidence rate in men than in women is that men may have more independent risk factors for CVDs than women, such as smoking, alcohol abuse, binge eating, and hypertension (44). In addition, postmenopausal women have higher levels of estrogen, which has an anti-atherosclerotic effect and plays a protective role in blood vessels (45). Maintaining a high dietary fiber intake may be beneficial for men.

Diabetes is a systemic disease that can lead to systemic vascular lesions. Myocardial metabolism disorders caused by diabetes can reduce the pumping function of myocardial cells, which is an important cause of HF. Dietary fiber can increase the sensitivity of peripheral tissues to insulin and improve insulin resistance, thereby reducing blood glucose levels. In addition, dietary fiber can reduce the activities of intestinal digestive enzymes and promote the repair of islet function (46). When the human body maintains a state of dyslipidemia for a long time, cholesterol in the blood may be deposited on the arterial wall to form atherosclerotic plaques and eventually lead to a variety of CVDs (47). Dietary fiber plays an important role in maintaining healthy blood lipids by binding to the bile and preventing cholesterol from being absorbed (48). For HF patients with a history of diabetes and dyslipidemia, a higher dietary fiber intake is a beneficial way to maintain their cardiovascular health.

Using the data from the NHANES database, we explored the association between dietary fiber intake and mortality among HF survivors. It is essential for clinicians, policymakers, and patients with HF to be aware of the benefits of dietary fiber for cardiovascular health management. In addition, it would be beneficial to add fiber-enriched foods to the diet of HF patients such as fresh fruits and vegetables, cereals, nuts, and legumes. However, our study has several limitations. First, as we used a retrospective cohort, we could not include all variables that might affect HF development. Second, the study had a limited sample size; however, the sufficient follow-up duration ensures a sufficient sample size for mortality outcome events. Third, due to the unavailability of NHANES data, we were unable to distinguish between types of dietary fiber. Previous studies have shown that dietary fiber from different sources has different compositions, structures, and rational properties and that its regulatory effects on body metabolism and intestinal flora are also different. Future studies need to consider the different types of dietary fiber when further exploring the association between dietary fiber and the prognosis of patients with HF (49). Finally, the NHANES database did not distinguish the degree of HF and whether the patients received surgical treatment was also unknown. Thus, the relationship between dietary fiber and different degrees of HF needs to be further explored.

## Conclusion

A high dietary fiber intake level was related to a lower risk of all-cause and CVD-caused mortality among HF survivors. Sufficient dietary fiber intake may be beneficial for maintaining heart health, especially in men, patients with a duration of HF ≥10 years, and those with a history of diabetes and dyslipidemia. Larger studies in the future are needed to evaluate the association between dietary fiber intake and mortality among HF patients.

## Data Availability

Publicly available datasets were analyzed in this study. This data can be found here: https://wwwn.cdc.gov/nchs/nhanes/.
