# Supplementary material for: Association between dietary fiber intake and all-cause and CVD-caused mortality among heart failure survivors: a cohort study from the NHANES database
Source: Front Cardiovasc Med. 2025 Apr 10;11:1406511. doi: 10.3389/fcvm.2024.1406511 (PMC12018438; doi:10.3389/fcvm.2024.1406511)
Supplement: Supplementary file 1 [file Table1.docx]

Table S1 Sensitivity analyses for data before and after imputation

| Variables | After imputation (n=1,510) | Before imputation (n=1,510) | Statistics | *P* |
| --- | --- | --- | --- | --- |
| BMI, kg/m^2^, n (%) |  |  | χ^2^=2.725 | 0.436 |
| <18.5 | 16 (0.89) | 14 (0.75) |  |  |
| 18.5-25 | 268 (16.95) | 255 (16.96) |  |  |
| 25-30 | 461 (28.98) | 436 (28.65) |  |  |
| ≥30 | 765 (53.17) | 729 (53.63) |  |  |
| Marital status, n (%) |  |  | χ^2^=2.874 | 0.719 |
| Married | 708 (49.81) | 703 (49.80) |  |  |
| Widowed | 389 (23.13) | 383 (23.06) |  |  |
| Divorced | 200 (13.06) | 198 (13.11) |  |  |
| Separated | 56 (3.14) | 55 (3.05) |  |  |
| Never married | 109 (7.91) | 108 (7.99) |  |  |
| Living with partner | 48 (2.96) | 48 (3.00) |  |  |
| Drinking, n (%) |  |  | χ^2^=3.246 | 0.197 |
| Never drinker | 595 (37.78) | 557 (37.25) |  |  |
| Regular drinker | 259 (18.98) | 247 (19.18) |  |  |
| Occasional drinker | 656 (43.24) | 628 (43.58) |  |  |
| Angina, n (%) |  |  | χ^2^=0.000 | 0.999 |
| No | 1119 (72.23) | 1094 (72.23) |  |  |
| Yes | 391 (27.77) | 381 (27.77) |  |  |
| Heart attack, n (%) |  |  | χ^2^=0.043 | 0.835 |
| No | 832 (55.14) | 825 (55.12) |  |  |
| Yes | 678 (44.86) | 672 (44.88) |  |  |
| Stroke, n (%) |  |  | χ^2^=0.189 | 0.664 |
| No | 1202 (80.10) | 1198 (80.09) |  |  |
| Yes | 308 (19.90) | 306 (19.91) |  |  |
| Uric acid, mg/dL, Mean (S.E) | 6.45 (0.07) | 6.42 (0.07) | t=1.84 | 0.067 |
| ALT, U/L, Mean (S.E) | 26.01 (1.85) | 26.10 (1.94) | t=-0.54 | 0.592 |
| AST, U/L, Mean (S.E) | 27.06 (1.00) | 27.14 (1.06) | t=-0.94 | 0.348 |
| Albumin, g/L, Mean (S.E) | 40.91 (0.13) | 40.87 (0.13) | t=1.09 | 0.276 |
| WBC, SI, Mean (S.E) | 7.75 (0.08) | 7.74 (0.08) | t=0.43 | 0.669 |
| Hemoglobin, g/dL, Mean (S.E) | 13.80 (0.07) | 13.79 (0.07) | t=1.21 | 0.230 |

t: t-test; χ^2^: chi-square; S.E: standard error;

BMI: body mass index; ALT: alanine aminotransferase; AST: aspartic transaminase; WBC: white blood cell.
